# Uncovering Hidden Transmission: Active Surveillance Reveals Cryptic Circulation of Yellow Fever Virus in Urban Marmosets in Belo Horizonte, Brazil, 2024

**DOI:** 10.3390/pathogens14090866

**Published:** 2025-09-01

**Authors:** Matheus Soares Arruda, Thaís Alkifeles Costa, Gabriel Dias Moreira, Daniel Jacob, Marcelle Alves de Oliveira, Mikaelly Frasson Biccas, Ana Maria de Oliveira Paschoal, Anna Catarina Dias Soares Guimarães, Samantha Stephany Fiuza Meneses Viegas, Gabriela Fernanda Garcia-Oliveira, Ana Luiza Campos Cruz, Letícia Trindade Almeida, Maria Fernanda Alves Souza e Silva, Daniel Ambrózio da Rocha Vilela, Thais Melo Mendes, Pedro Augusto Alves, Kathryn A. Hanley, Nikos Vasilakis, Marina do Vale Beirão, Betânia Paiva Drumond

**Affiliations:** 1 Laboratório de Vírus, Instituto de Ciências Biológicas, Universidade Federal de Minas Gerais, Belo Horizonte 31270-901, MG, Brazil; matheusmtsa095@gmail.com (M.S.A.); alkifelescosta@hotmail.com (T.A.C.); gabriel.dias05082000@gmail.com (G.D.M.); danieljacob.bio@gmail.com (D.J.); marcelle.oliveira@ufv.br (M.A.d.O.); mfrassont@gmail.com (M.F.B.); anamuzenza@gmail.com (A.M.d.O.P.); annacatarinaanna@gmail.com (A.C.D.S.G.); samfviegass@gmail.com (S.S.F.M.V.); gabrielafernandag@gmail.com (G.F.G.-O.); ana.luiza.campos.cruz@gmail.com (A.L.C.C.); 2 Instituto René Rachou-Fundação Oswaldo Cruz, Belo Horizonte 30190-002, MG, Brazil; leticia.trindade.almeida@gmail.com (L.T.A.); mfmariafernandas@gmail.com (M.F.A.S.e.S.);; 3 Instituto Brasileiro do Meio Ambiente e dos Recursos Naturais Renováveis, Belo Horizonte 30110-051, MG, Brazil; daniel.vilela@ibama.gov.br; 4 Diretoria de Zoonoses da Prefeitura de Belo Horizonte, Belo Horizonte 30110-051, MG, Brazil; thaismelo.mendes@pbh.gov.br; 5 Department of Biology, New Mexico State University, Las Cruces, NM 88003, USA; khanley@nmsu.edu; 6 Department of Pathology, University of Texas Medical Branch, Galveston, TX 77555-0609, USA; nivasila@utmb.edu; 7 Center for Vector-Borne and Zoonotic Diseases, The University of Texas Medical Branch, Galveston, TX 77555-0609, USA; 8 Institute for Human Infection and Immunity, University of Texas Medical Branch, Galveston, TX 77555-0610, USA

**Keywords:** non-human primates, mosquito, vectors, yellow fever virus, sylvatic yellow fever, active surveillance, cryptic circulation

## Abstract

Between 2016 and 2018, the state of Minas Gerais, Brazil, experienced its most significant yellow fever (YF) outbreak in 80 years. Yellow fever virus (YFV) circulation persisted afterward, with continued non-human primate (NHP) epizootics and, recently, human cases. In June 2024, YFV RNA was detected in a dead marmoset (*Callithrix penicillata*) in an urban square in Belo Horizonte (BH), prompting a field investigation in an adjacent park to assess infection in potential mosquito vectors and NHPs. A total of 250 mosquitoes representing nine species were collected at ground and canopy level, of which *Aedes fluviatilis* and *Aedes scapularis* comprised 78.8% of the specimens. *Haemagogus* spp. and *Sabethes* spp. mosquitoes were not collected, possibly due to the short sampling window during the dry season. No active YFV infection was detected in any of the mosquito pools tested. Eight marmosets (*Callithrix penicillata*) were captured and tested for arboviral infections. Five out of eight sera, representing both adult and juvenile (less than 17 months old) animals, tested positive for anti-YFV IgM. Interestingly, two adults recaptured in later expeditions revealed seroconversion. One was IgM-positive in July 2024 but negative by September 2024, consistent with the expected decline in IgM levels. The other, initially IgM-negative (as of July 2024), tested positive in April 2025, indicating recent exposure to YFV. These findings provide evidence for the ongoing, low-level circulation of YFV among urban NHPs, posing a continued risk of viral spillover to humans. Moreover, these results highlight the importance of active surveillance in detecting recent infections that would likely be missed by passive monitoring. This integrated approach enhances our understanding of local YF epidemiology and supports early, evidence-based public health interventions to prevent future human outbreaks.

## 1. Introduction

In late 2016, yellow fever virus (YFV) (*Orthoflavivirus flavi, Flaviviridae*) reemerged in southeastern Brazil, leading to major human outbreaks [1]. The state of Minas Gerais (MG) was the most heavily affected between 2016 and 2018, with 1006 confirmed human cases and 340 reported deaths [2,3]. Following this period, increased vaccination coverage contributed to a decrease in new human cases [4]. Nevertheless, YFV has continued to circulate in the region. Since 2020, epizootics in non-human primates (NHPs) and sporadic human cases have been reported in MG [5,6,7,8,9]. In MG, the most recent confirmed human fatality due to yellow fever (YF) occurred in December 2024 in the southern region of the state, with 16 epizootics being reported in the region in 2025 [10].

Monitoring YFV in NHPs is a key strategy for identifying areas of ongoing viral circulation, enabling targeted public health interventions to prevent human infections. This strategy is one of the pillars of the Brazilian Program for YF surveillance [11]. BH contains numerous urban parks that are inhabited by NHPs, primarily marmosets (*Callithrix penicillata*), which have adapted to urban environments and frequently interact with humans [12]. Furthermore, sylvatic mosquito vectors of YFV (*Haemagogus* spp. and *Sabethes* spp.) have been previously identified in forested areas surrounding BH. Given the extensive forest fragmentation is associated with urban development, these vectors may also be present in urban parks and green spaces within the urban landscape [12,13,14]. The co-occurrence of NHPs, sylvatic mosquito vectors, and humans within urban green areas poses a risk to the maintenance and spillover of sylvatic YFV transmission and spillover within BH.

**Figure 1:**
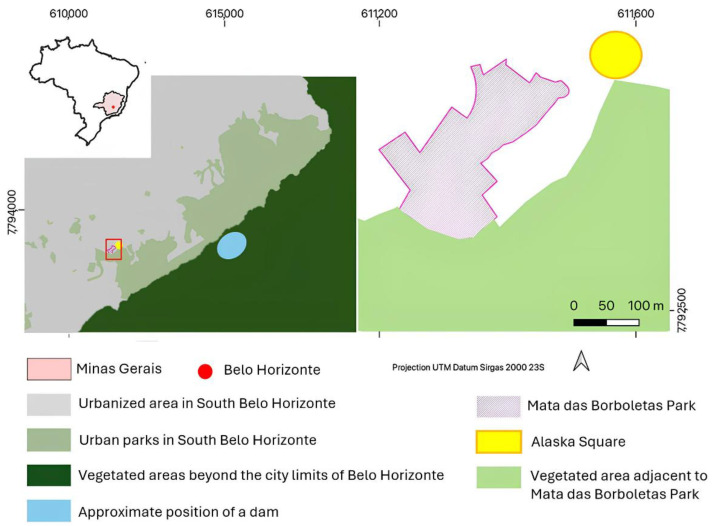
Map showing the location of Mata das Borboletas Park in Belo Horizonte, Brazil. From left to right: (i) a smaller map of Brazil showing the location of Minas Gerais state (pale pink) and city of BH (red); (ii) a view of the southern part of BH, with the boundaries of Mata das Borboletas Park outlined in purple; (iii) a detailed view of the study area, where the yellow circle indicates Alaska Square where a *Callithrix* sp. was found dead and infected with YFV in June 2024, and the purple outline indicates Mata das Borboletas Park. The map was generated using QGIS, a free open source (GNU General Public License) system developed by OSGeo (Open Source Geospatial Foundation) (https://www.qgis.org, accessed on 3 August 2025). The geographic data presented in the map was obtained from the Brazilian government database (https://www.ibge.gov.br/geociencias/organizacao-do-territorio/malhas-territoriais/15774-malhas.html?=&t=acesso-ao-produto, accessed on 3 August 2025) and the Belo Horizonte city hall database (https://bhmap.pbh.gov.br/, accessed on 3 August 2025).

In June 2024, YFV infection was confirmed in a *C. penicillata* carcass collected in a green square (Alaska Square, Figure 1) in the southern region of BH [15]. In response, in collaboration with Zoonoses Department of BH, surveillance efforts were launched to monitor YFV circulation through the collection of mosquitoes and NHPs in the nearest urban park, 100 m away from the green square (Figure 1). This study aimed to investigate YFV infection in mosquitoes and NHPs sampled in this urban park of BH.

## 2. Materials and Methods

### 2.1. Study Area

This study was conducted in Mata das Borboletas Park, a 34,600-square-meter park area situated within a densely populated neighborhood in the southern region of BH (Figure 1), which serves as a leisure spot for locals. It contains two river sources, and 80% of its area is covered by Cerrado and Atlantic Forest tree species, as well as some exotic species, i.e., *Pleioblastus variegatus* [16]. A busy roadway separates the park from an adjacent green corridor that connects it to Mangabeiras Park, the largest park in southern BH, which borders a forested area on the outskirts of the city (Figure 1).

### 2.2. Mosquito Sampling and Identification

Mosquito collections were carried out under SISBIO permit 77400-8 (approved on 18 June 2024). Fieldwork was conducted over three consecutive days, from 29 July–1 August 2024, during the dry season in BH. Three points, 90 to 100 m apart from each other, were selected considering (*i*) the park’s limited size; (*ii*) paved areas (around 20% of the park’s area), which constrained suitable locations for trap placement; (*iii*) sufficient distance between points to reduce overlap and ensure sampling independence; and (*iv*) human activity in order to avoid interference in the collection. BG-Sentinel^®^ (Clarke, Roselle, IL, USA) traps baited with dry ice and BG-Lure^®^ (Clarke, Roselle, IL, USA) were placed in the tree canopy (approximately 5 m high) and at ground level. Each trap was equipped with a temperature data logger (Kestrel DROP D1, Kestrel Instruments, Minneapolis, MN, USA). Mosquitoes were removed from the traps, and dry ice was replenished every morning (around 7 am) and evening (around 4 pm).

Additionally, manual collections, using hand nets and aspirators, were conducted for two consecutive days in each of the three sampling points described above. Manual mosquito collections were conducted in two daily shifts: one in the morning (7 am) and one in the afternoon (4 pm). Hand nets were used by collectors to catch foraging mosquitoes actively, both in the canopy (using a ladder) and at ground level, during 30 min shifts, while entomological aspirators were also used for 15 min on each shift. The collectors were rotated for each site and day to eliminate collection bias.

Mosquito specimens were placed in 15 mL conical tubes, stored on dry ice, and transported to the laboratory for identification and processing. Species-level identification was initially performed using a stereomicroscope and standard taxonomic keys in the lab [17]. For identification, mosquitoes were placed on a homemade cold table, created by placing a Petri dish over dry ice. To avoid cross-contamination, individual batches were not manipulated with the same utensils, and surfaces were constantly cleaned during the identification. In some cases, molecular identification was also conducted using DNA barcoding. Total DNA was extracted from mosquito legs using the Wizard^®^ Genomic DNA Purification Kit (Promega, Madison, WI, USA), and the mitochondrial cytochrome C oxidase subunit I (COI) gene was amplified by PCR, following the protocol described by Hebert and colleagues [18] (Table 1).

Amplicons were sequenced using the Sanger method [25] in collaboration with Instituto René Rachou/Fiocruz Minas (ABI 3730XL, Applied Biosystems, Thermo Fisher Scientific, Waltham, MA, USA). Raw sequence data were analyzed using a graphical tool designed here to assemble high-quality consensus sequences from forward and reverse Sanger reads (for details see Appendix A). The software, called Samanthex 2.0, integrates alignment, base quality assessment, and consensus generation, allowing users to apply Phred quality score thresholds to ensure data reliability. Samanthex also facilitates BLAST searches directly from the interface against the NCBI database. The tool is open-source and freely available at GitHub [26] and Zenodo [27]. Final consensus sequences were compared with reference sequences in the NCBI GenBank and the Barcode of Life Data System [28] for species confirmation based on sequence similarity.

NHPs were captured during three field expeditions conducted at Mata das Borboletas Park between July 2024 and April 2025. Two expeditions took place during the dry season, the first on 29 July 2024, and the second on 2, 4 and 6 September 2024. The third expedition took place during the wet season, on 14 April 2025. Fresh fruits were used to attract and bait animals to Tomahawk live traps. Once trapped, each NHP was contained and sedated with a solution consisting of 5% midazolam (0.1 mg/kg) and 10% ketamine (10 mg/kg). All NHPs were microchipped, and their age classification was determined based on physical characteristics, including fur coloration, presence of ear tufts, body weight and size, and dental condition, following the criteria established by Yamamoto and colleagues [29]. Age classification was performed as follows: infants (0–5 months) lack of ear tufts and forehead marking; fur is sparse on chest and abdomen; juveniles (6–17 months) present partial ear tuft and forehead patch development, with deciduous tooth replacement; adults (>17 months) have fully developed ear tufts and forehead marking; and old adult individuals display same characteristics as adults plus dental wear or loss, and tartar accumulation [29].

Up to 1% of the total body weight in blood was collected from each NHP by veterinarians. Once an NHP had fully regained consciousness, it was released at the collection site, on the same day, before dawn. The blood samples were kept in a thermal box at room temperature during fieldwork and transported to the laboratory within a maximum of four hours, where they were centrifuged at 3500× *g* for 15 min to separate the serum, which was stored at −80 °C until testing.

### 2.3. YFV Molecular Screening

Total RNA was extracted from the 10 serum samples (140 µL) obtained from eight *C. penicillata* individuals (two of whom were captured twice, while the remaining six were captured only once) using the QIAamp Viral RNA Mini Kit (QIAGEN, Germantown, MD, USA). A negative control, consisting only of reagents, was included in each extraction batch. The extracted RNA was first tested by RT-qPCR for the presence of the endogenous β-actin gene [19], serving as an internal control. Subsequently, samples were screened for the 5′ UTR region of the YFV genome [20], the NS5 gene of Zika virus (ZIKV) [21], the S segment of OROV [22], and the E1 gene of CHIKV [23] using specific primers and probes. Additionally, samples were tested to detect the NS5 region of the orthoflavivirus’ genome [24] (Table 1).

For viral detection in mosquitoes, pools of up to 10 individuals of the same species and sampling stratum were placed in 2.0 mL screw-cap tubes containing borosilicate beads and 450 µL of minimum essential medium (MEM) supplemented with 2% fetal bovine serum (FBS) and 2% HEPES. Samples were homogenized using an automated tissue homogenizer (Mini-Beadbeater-16, BioSpec, Bartlesville, OK, USA) and centrifuged at 16,000× *g* for 3 min. A volume of 250 µL of supernatant was collected and stored (to be used for viral isolation in case of viral positive samples). In contrast, the remaining material was used for total RNA extraction with the RNeasy Mini Kit (QIAGEN, Germantown, MD, USA). Up to 13 pools, plus the negative extraction control, were extracted at once. Mosquito RNA samples were screened by RT-qPCR targeting the NS5 region of the orthoflavivirus’ genome [24] and the 5′ UTR region of the YFV genome [20].

Amplification was performed in all assays using the GoTaq^®^ Probe 1-Step RT-qPCR System (Promega, Madison, WI, USA), with the addition of SYBR Green (for β-actin and orthoflavivirus RTq-PCR) or the use of a specific probe (for YFV, ZIKV, OROV, and CHIKV RTq-PCR). Each RT-qPCR assay included a negative extraction control, a non-template control, and a positive control.

### 2.4. Serological Screening

NHP sera were tested for anti-YFV IgM antibodies using a commercial rapid lateral flow immunoassay (Febre Amarela IgM ECO Teste, EcoDiagnóstica, Corinto, MG, Brazil), following the manufacturer’s protocol. Briefly, 10 µL of each serum was mixed with the provided buffer and added to test cassettes containing YFV-specific antigens. The cassettes were incubated at room temperature for up to 15 min, and results were interpreted based on the appearance of a colored band indicating reactivity. A biobanked YFV-positive human serum was included as positive control. Biobanked sera from a *C. penicillata,* presenting neutralizing antibodies against YFV were also used as a positive control in IgM test. To assess potential cross-reactivity among orthoflavivirus antibodies, YFV IgM-positive samples were further tested using rapid lateral flow assays for the detection of IgM and IgG antibodies against dengue (DENV) and ZIKV viruses (DENV IgG/IgM Premium EcoTeste and Zika IgG/IgM ECO Teste, EcoDiagnóstica, Corinto, MG, Brazil).

A plaque reduction neutralization test (PRNT) was conducted using the YFV 17DD virus, serum samples were heat-inactivated at 56 °C for 30 min to eliminate complement system activity. Subsequently, samples were diluted two-fold from 1:5 to 1:40 and homogenized with a viral suspension containing approximately 100 plaque-forming units (PFU/mL) and incubated at 37 °C for 1 h. Following this incubation, 90 µL of the sample-virus mixture was inoculated onto Vero cell monolayers. Adsorption occurred for 1 h, with plates agitated every 10 min. Finally, 1% carboxymethylcellulose (CMC) (Labsynth, Diadema, SP, Brazil) overlay was added to each well, and plates were incubated at 37 °C with 5% CO_2_ for 5 days. Plates were then fixed with 3.7% formaldehyde (Sciavicco, São Paulo, SP, Brazil) for 1 h, washed, and stained with 1% crystal violet (Newprov, Pinhais, PR, Brazil). After a final wash, PFUs were counted to calculate the percentage of viral neutralization. Serum from uninfected mice was used as a negative control and serum from mice experimentally infected with YFV as a positive control (Supplementary Figure S1). The cutoff of 50% of neutralization (PRNT_50_) on dilution 1:10 was considered for positivity.

## 3. Results and Discussion

We collected 250 mosquitoes belonging to nine taxa (Table 2). The most abundant species was *Aedes fluviatilis*, with 123 individuals, accounting for 49.2% of the sample. Most of the mosquitoes, 85.6%, were sampled at ground level, indicating an increased possibility of contact between mosquito vectors and humans in this stratum. We were able to identify 243 females and five males. None of the females was engorged. Mosquitoes were separated into 39 pools, all of which were negative by RT-qPCR for both the detection of orthoflavivirus NS5 and CHIKV E1 genome regions. Despite the absence of YFV-positive pools, the collection included 94 mosquitoes belonging to species recognized as potential YFV vectors, specifically *Ae. albopictus* (n = 14), *Ae. aegypti* (n = 6), and *Ae. scapularis* (n = 74) [30,31,32]. The absence of *Haemagogus* spp. and *Sabethes* spp. in our collections should not be taken as evidence that these sylvatic vectors are ecologically absent from the park. Rather, it likely reflects confinement of our sampling strategy to the dry season, a period when mosquito populations and densities are naturally low. To more rigorously assess the presence and seasonal dynamics of sylvatic vectors in this area, future studies should include longitudinal sampling across both dry and rainy seasons with increased spatial coverage.

A total of eight *C. penicillata* individuals were captured, and none showed visible clinical signs of disease at the time of sampling. During the first expedition (July 2024), seven individuals were sampled (two juveniles, four adults, and one old adult) (Table 3). In the second expedition (September 2024), one adult (CT24-216) previously captured in July 2024 was recaptured and resampled. The third expedition (April 2025) yielded two *C. penicillata,* one old adult and one adult (CT24-214), which had also been sampled during the first expedition (Table 3).

None of the animals tested positive by RT-qPCR for any of the arboviruses investigated, indicating no active infection at the time of sampling. However, five *C. penicillata*, including both adults and juveniles, exhibited faint bands for YFV IgM in a rapid lateral flow assay and were classified as positive according to the manufacturer’s instructions (Supplementary Figure S2). All YFV IgM-positive sera tested negative for anti-DENV and anti-ZIKV IgM/IgG, ruling out cross-reactivity and supporting a recent and specific exposure to YFV (Supplementary Figure S2). To further investigate the presence of neutralizing antibodies, PRNT assays were performed against the YF-17DD strain. None of the IgM-positive animals reached the 50% neutralization threshold, being all negative (Table 3, Supplementary Table S1).

Although neutralizing antibodies are considered the gold standard for confirming YFV infection, a previous study showed that NHP exposed to mosquitoes infected with DENV and ZIKV, despite not developing neutralizing antibodies against these viruses, produced low levels of binding (IgG) antibodies [33]. This pattern is similar to that observed in the present study, in which IgM antibodies were detected but neutralizing antibodies were absent in the NHPs studied here. Previous studies in southeastern Brazil have demonstrated that during interepidemic periods, YFV genomic loads in NHPs were significantly lower than those recorded during epidemic peaks (2017–2018) in MG [8,34]. Similar observations were recorded during post-outbreak surveillance in the state [6], as was the case of the NHP sampled in June 2024 in Alaska Square, near Mata das Borboletas Park (Thais Mello, pers. comm.). Such low-level viral exposures may induce insufficient immune stimulation to elicit a neutralizing antibody response, while still allowing the induction and detection of binding non-neutralizing antibodies [33,35]. Previous studies have shown that neutralizing antibody levels can vary according to the infectious dose. Mason and colleagues [36] found a positive dose-response regarding neutralizing antibodies in rhesus macaques vaccinated with serial dilutions of YF-17D. Hanley and colleagues [37] reported higher viremia and stronger neutralizing responses in mice bitten by more ZIKV-infected mosquitoes. However, recent studies indicate that higher viral doses do not always translate into higher neutralizing antibody levels, revealing more complex immunological interactions. For example, in squirrel monkeys, higher ZIKV doses produced peak titers exceeding those of DENV-2, yet PRNT_80_ values were significantly lower, contrasting with the stronger neutralizing responses elicited by lower-dose DENV-2 infection [38].

While it is known that IgM antibodies can persist for extended periods following orthoflavivirus infections [39,40], the presence of anti-YFV IgM in juvenile *C. penicillata*, estimated to be from 6 to 17 months old, born after the 2016–2018 YF outbreaks in MG, suggests the occurrence of recent viral transmission within the past two years. The age determination of NHPs was based on morphological features and, as such, subject to some degree of uncertainty due to inter-individual variation and observer interpretation. Nonetheless, the detection of IgM in adults makes it unlikely that these antibodies represent residual responses from past epidemic exposure (2016–2018). Among the eight sampled marmosets, two were recaptured. Animal CT24-214 was IgM-positive in July 2024 and negative in September 2024, consistent with the expected decline in IgM levels [41]. Conversely, the animal CT24-216 was IgM-negative in July 2024 and positive in April 2025, although negative in PRNT_50_, indicating a new exposure within this interval. Altogether, these findings provide evidence of continued YFV circulation in the BH metropolitan area, eight years after the last human outbreaks in the state began in 2016 [3], with recent surveillance data reinforcing these findings [6,15].

The detection of YFV IgM-positive *C. penicillata* in Mata das Borboletas Park, combined with the park’s proximity to larger forest fragments, underlines the potential risk of YFV dissemination from sylvatic zones into urban environments via this ecological corridor. The identification of *Ae. scapularis,* one of the predominant species in our mosquito collection, and *Ae. albopictus*, both of which exhibit sylvatic behaviors, further raise concerns about the possibility of YFV spillover into urban settings. These species are highly adaptable, being found in peridomestic environments feeding on a range of hosts including humans, and can be competent vectors of YFV, enhancing their role as potential bridge vectors between sylvatic and urban environments [31,32]. These findings reinforce the critical need for sustainable entomological and NHP surveillance in areas considered non-endemic for YF. The coexistence of sylvatic and urban vectors and vertebrate hosts in major Brazilian cities [11,42,43] highlights a persistent risk for both the maintenance of the sylvatic cycle and the potential reestablishment of urban YF. Continued monitoring and the integration of public health strategies, such as increasing vaccination in peri-urban areas and promoting educational activities for the community, are essential to prevent future outbreaks in these densely populated areas.

## 4. Conclusions

The detection of anti-YFV IgM in *C. penicillata* captured in an urban park coupled with the death of another individual infected with YFV in BH in mid-2024 provides evidence of recent YFV circulation in low levels in a densely populated area of southeastern Brazil. These findings emphasize the continued vulnerability of urban areas to YFV reintroduction, even several years after the last major human outbreaks. Strengthened entomological and virological surveillance in the metropolitan region of BH and other major cities is urgently needed.

## Figures and Tables

**Table 1:** Primers and probes used for investigating arboviruses and hosts in the sampled mosquitoes at Mata das Borboletas Park, BH, Brazil.

Target	Sequence	Reference
COI	5′-GGTCAACAAATCATAAAGATATTGG-3′ (F) 5′-TAAACTTCAGGGTGACCAAAAAATCA-3′ (R)	Hebert et al., 2003 [18]
β-actin	5′-CCAACCGCGAGAAGATGA-3′ (F) 5′-CCAGAGGCGTACAGGGATAG-3′ (R)	Rezende et al., 2019 [19]
YFV	5′-GCTAATTGAGGTGYATTGGTCTGC-3′ (F) 5′-ATCGAATGCACCGCACACT-3′ (R) 5′-ATCGAGTTGCTAGGCAATAAACAC-3′ (P)	Domingo et al., 2012 [20]
ZIKV	5′-CCGCTGCCCAACACAAG-3′ (F) 5′-CCACTAACGTTCTTTTGCAGACAT-3′ (R) 5′-AGCCTACCTTGACAAGCAGTCAGACACTCAA-3′ (P)	Lanciotti et al., 2008 [21]
OROV	5′ TCCGGAGGCAGCATATGTG-3′ (F) 5′-ACAACACCAGCATTGAGCACTT-3′ (R) 5′-CATTTGAAGCTAGATACGG-3′ (P)	Naveca et al., 2017 [22]
CHIKV	5′-TCGACGCGCCCTCTTTAA-3′ (F) 5′-CTGCTAATCGCTCAAMGAACG-3′ (R) 5′-ACCAGCCTGCACCCATTCCTCAGAC-3′ (P)	Edwards et al., 2007 [23]
Pan-orthoflavivirus	5′-TACAACATGATGGGGAARAGAGARAA-3′ (F) 5′-GTGTCCCAGCCNGCKGTGTCATCWGC-3′ (R)	Patel et al., 2013 [24]

Primers are indicated by the letters F (forward) and R (reverse). Probes, when present, are indicated by the letter P. COI: cytochrome C oxidase subunit I; YFV: yellow fever virus; ZIKV: Zika virus; OROV: Oropouche virus; CHIKV: chikungunya virus.

**Table 2:** Mosquito taxa collected by stratum in Mata das Borboletas Park, BH, Minas Gerais, 2024.

Genus/species	Ground	Canopy	Total
*Aedes aegypti*	5	1	6
*Aedes albopictus*	13	1	14
*Aedes fluviatilis*	112	11	123
*Aedes scapularis*	66	8	74
*Aedes* spp.	3	0	3
*Culex* spp.	9	13	22
*Wyeomyia* spp.	1	0	1
*Wyeomyia melanocephala*	3	2	5
Total	212	36	248 *

* Two pools containing one individual each could not be identified by barcoding.

**Table 3:** *Callithrix penicillata* captured and tested using molecular and serologic methods at Mata das Borboletas Park, Belo Horizonte, Brazil.

NHP ID	Month	Age	RT-qPCR					PRNT_50_	Lateral Flow Test		
			YFV	CHIKV	ZIKV	OROV	Pan Flavi		YFV IgM	DENV IgM/ IgG	ZIKV IgM/ IgG
CT24-214	July 2024	adult	neg	neg	neg	neg	neg	neg	pos	neg/ neg	neg/ neg
CT24-215	July 2024	adult	neg	neg	neg	neg	neg	neg	neg	na	na
CT24-216	July 2024	adult	neg	neg	neg	neg	neg	neg	neg	na	na
CT24-217	July 2024	juvenile	neg	neg	neg	neg	neg	neg	pos	neg/ neg	neg/ neg
CT24-218	July 2024	juvenile	neg	neg	neg	neg	neg	neg	pos	neg/ neg	neg/ neg
CT24-219	July 2024	adult	neg	neg	neg	neg	neg	neg	pos	neg/ neg	neg/ neg
CT24-220	July 2024	old adult	neg	neg	neg	neg	neg	neg	neg	na	na
CT24-216 *	September 2024	adult	neg	neg	neg	neg	neg	neg	pos	neg/ neg	neg/ neg
CT24-234	April 2025	old adult	neg	neg	neg	neg	neg	neg	neg	na	na
CT24-214 *	April 2025	adult	neg	neg	neg	neg	neg	neg	neg	na	na

NHP ID: non-human primate identification. Month: month of NHP sampling. YFV: yellow fever virus. CHIKV: chikungunya virus. ZIKV: Zika virus. OROV: Oropouche virus. Pan Flavi: pan-orthoflavivirus. PRNT_50_: plaque reduction neutralization test with cutoff of 50%. IgM: immunoglobulin M. DENV: Dengue virus. IgG: immunoglobulin G. Samples with less than 50% of neutralization on dilution 1:10 were considered negative. (*) indicate animals that were recaptured and resampled. pos: positive; neg: negative; na: not available.

## Data Availability

All research data are shared within the manuscript and in the ZENODO repository under DOI 10.5281/zenodo.16052796 (deposited on 10 July 2025).

## Supplementary Materials

The following supporting information can be downloaded at: https://www.mdpi.com/article/10.3390/pathogens14090866/s1, Figure S1: Representative plates of YFV PRNT performed with NHP serum. The pictures show 24 well plates from PRNT performed with samples 214–218 (A) and controls (B) consisting of sera obtained from mice infected with YFV 17DD and mock-infected mice. All samples were negative, i.e., did not reach 50% of neutralization. CV: virus control; CC: cell control; POS: seropositive control; NEG1 and NEG2: seronegative control. Figure S2: Results of rapid lateral flow tests to investigate YFV IgM and ZIKV and DENV IgM and IgG in sera from non-human primates collected in the Mata das Borboletas park. A-E: results of samples which were positive in YFV IgM tests and negative in DENV or ZIKV IgM/IgG tests [(214 (fig. A), 216R (fig. B), 217 (fig. C), 218 (fig. D), 219 (fig. E)]. F: results of samples that tested negative for YFV IgM (215, 216, 220, 214R and 234). G: results of positives control from left to right: human biobanked serum, serum from *C. penicilatta* (previously positive in plaque reduction neutralization test using YFV 17DD: 57% neutralization in the 1:20 dilution), biobanked human sera used for DENV and ZIKV tests, respectively.; Table S1: Percentage of YFV 17DD neutralization values obtained in PRNTs using sera from *C. penicillata* collected in the Mata das Borboletas park, BH, MG, 2024 and 2025.

## Abbreviations

The following abbreviations are used in this manuscript:

µL	microliter
BH	Belo Horizonte
CEUA	Ethical Committee for Animal Experimentation
CHIKV	chikungunya virus
CMC	carboxymethylcellulose
COI	cytochrome c oxidase subunit I
DNA	deoxyribonucleic acid
E1	envelope protein 1
F	forward
HEPES	2-[4-(2-hydroxyethyl)piperazin-1-yl]ethanesulfonic acid
ID	identification
IgG	immunoglobulin G
IgM	immunoglobulin M
kg	kilogram
MEM	minimum essential medium
MG	Minas Gerais state
mg	milligram
mL	milliliter
n	number
na	not available
NCBI	National Center for Biotechnology Information
neg	negative
neut	neutralization
NHP	non-human primate
NS5	nonstructural protein 5
°C	Celsius degrees
OROV	Oropouche virus
P	probe
PCR	polymerase chain reaction
PFU	plaque-forming units
pos	positive
PRNT	plaque-reduction neutralization test
R	reverse
RNA	ribonucleic acid
RT-qPCR	reverse transcriptase polymerase chain reaction
SISBIO	Brazil’s Biodiversity Licensing and Information System
UTR	untranslated region
YF	yellow fever
YFV	yellow fever virus
ZIKV	Zika virus

## Appendix A

To generate high-fidelity consensus sequences from experimental Sanger sequencing replicates, we developed a custom graphical tool, Samanthex 2.0. The program was implemented in Python 3, utilizing the Tkinter library for the graphical user interface and the BioPython 1.85 library for all bioinformatic operations. The development of the Samanthex codebase was supported by generative AI; GPT-4 large language model was utilized under the direct supervision of the authors to assist in debugging, code optimization, and algorithmic revision.

The workflow begins with the user providing multiple forward and reverse sequencing reads (.ab1 or .phd.1 formats), which are treated as technical replicates. For each read, the program extracts the nucleotide sequence and its corresponding Phred quality scores. All reverse reads undergo a critical pre-processing step wherein the sequence is reverse-complemented and its quality score profile is inverted to match the new 5′ to 3′ orientation.

To consolidate the information from multiple replicates, Samanthex first constructs a consensus contig for all forward reads and, separately, for all reverse reads, through a progressive alignment heuristic. This process begins with the first replicate sequence as a provisional consensus, to which subsequent replicates are iteratively aligned using the Bio.pairwise2.align.localms function. A new consensus is generated at each step based on quality-driven logic: if aligned bases match, the base is retained with the maximum of the two quality scores; for mismatches, the base with the higher Phred score is selected; and for insertions or deletions, the existing base is carried over. Following the generation of the master forward and reverse contigs, they are aligned against each other using the same methodology to produce the final consensus sequence. A user-defined minimum Phred score is enforced as a final quality control measure, masking any base with a quality score below this threshold as an ‘N’. The final high-fidelity sequence is exported in FASTA format, and the tool additionally provides an integrated function to submit the sequence directly to a BLASTn search against the NCBI database for immediate identification.
