# Supplementary material for: Uncovering Hidden Transmission: Active Surveillance Reveals Cryptic Circulation of Yellow Fever Virus in Urban Marmosets in Belo Horizonte, Brazil, 2024
Source: Pathogens. 2025 Sep 1;14(9):866. doi: 10.3390/pathogens14090866 (PMC12472655; doi:10.3390/pathogens14090866)
Supplement: Supplementary file 1 [file pathogens-14-00866-s001.zip › pathogens-3801880-supplementary.pdf]

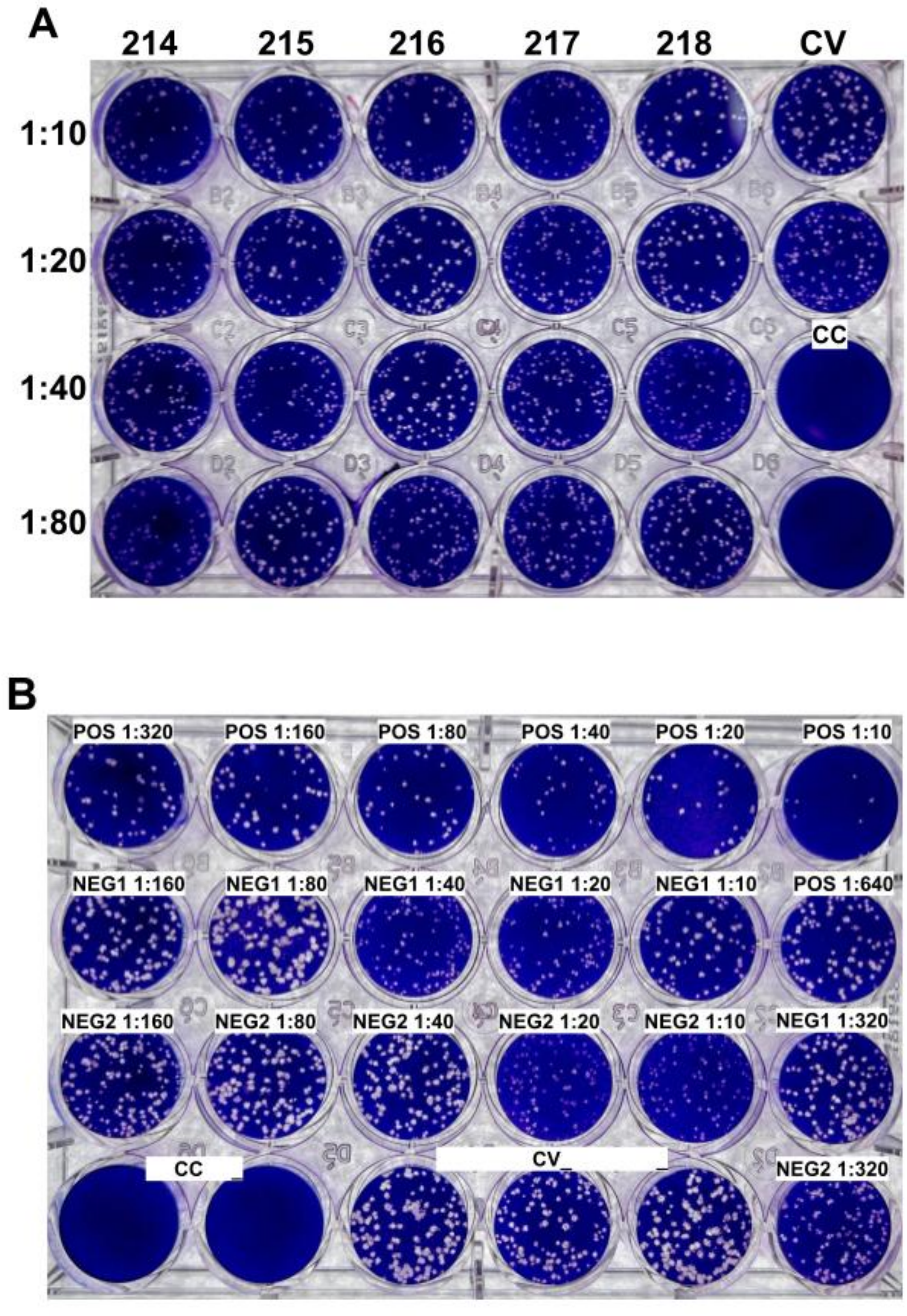

Sup Fig S1. Representative plates of YFV PRNT performed with NHP serum. The pictures show 24-well plates from PRNT performed with samples 214-218 (A) and controls (B) consisting of sera obtained from mice infected with YFV 17DD and mock-infected mice. All samples were negative, i.e. did not reach 50% of neutralization. CV: virus control; CC: cell control; POS: seropositive control; NEG1 and NEG2: seronegative control.

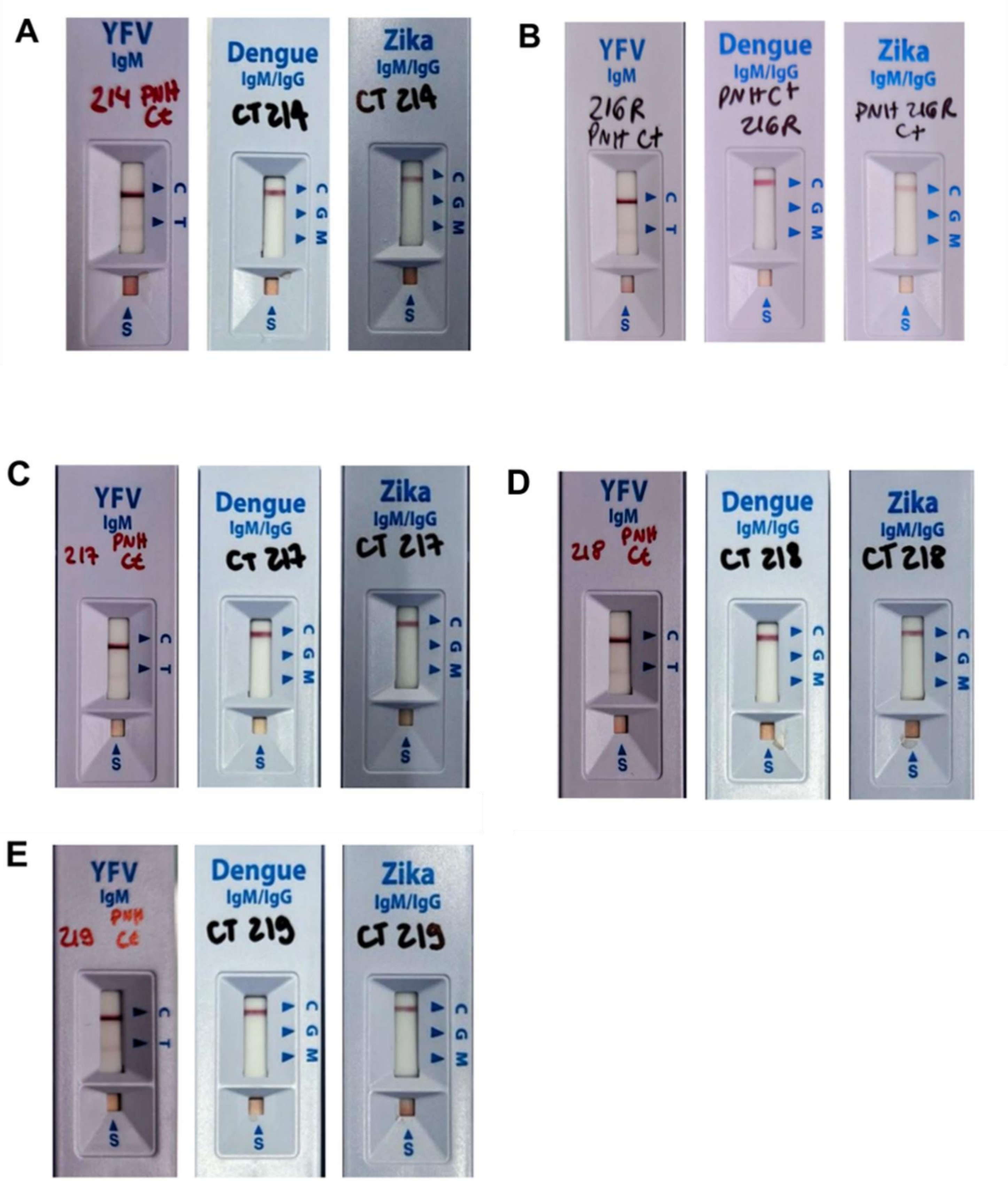
A
YFV
IgM
214 PNH Ct
C
T
S
Dengue
IgM/IgG
CT 214
C
G
M
S
Zika
IgM/IgG
CT 214
C
G
M
S
B
YFV
IgM
216R PNH Ct
Dengue
IgM/IgG
PNH Ct 216R
Zika
IgM/IgG
PNH 216R Ct
C
D
YFV
IgM
217 PNH Ct
Dengue
IgM/IgG
CT 217
Zika
IgM/IgG
CT 217
D
YFV
IgM
218 PNH Ct
Dengue
IgM/IgG
CT 218
Zika
IgM/IgG
CT 218
E
YFV
IgM
219 PNH Ct
Dengue
IgM/IgG
CT 219
Zika
IgM/IgG
CT 219

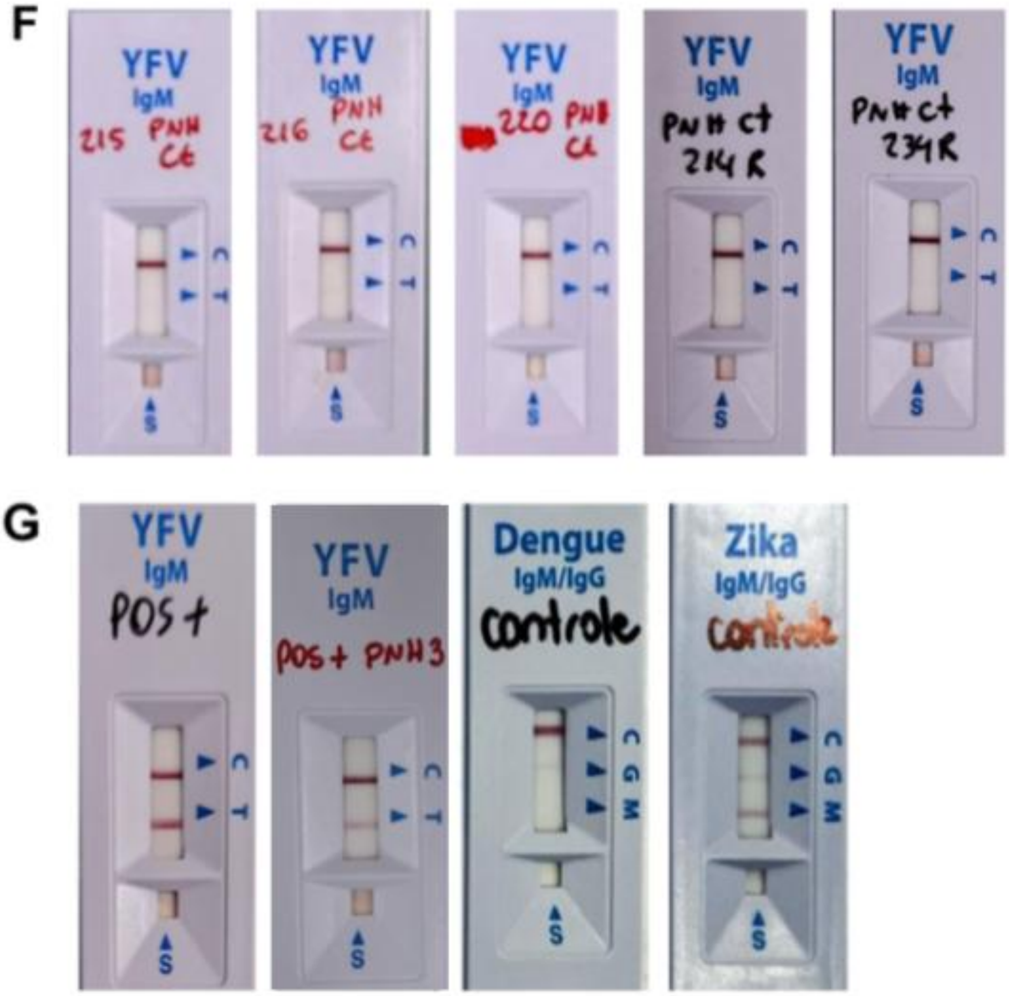

Sup Fig S2: Results of rapid lateral flow tests to investigate YFV IgM and ZIKV and DENV IgM and IgG in sera from non-human primates collected in the Mata das Borboletas park. A-E: results of samples which were positive in YFV IgM tests and negative in DENV or ZIKV IgM/IgG tests [(214 (fig. A), 216R (fig. B), 217 (fig. C), 218 (fig. D), 219 (fig. E)]. F: results of samples that tested negative for YFV IgM (215, 216, 220, 214R and 234). G: results of positives control from left to right: human biobanked serum, serum from *Callithrix penicilatta* (previously positive in plaque reduction neutralization test using YFV 17DD: 57% neutralization in the 1:20 dilution), biobanked human sera used for DENV and ZIKV tests, respectively.

Sup Table S1: Percentage of yellow fever virus 17DD neutralization values obtained in plaque reduction neutralization tests using sera from *Callithrix penicillata* collected in the Mata das Borboletas park, Belo Horizonte, Minas Gerais, 2024 and 2025.

	Dilution Neutralization (%)			
NHP ID	1:10	1:20	1:40	1:80
CT24-214	48.2	24.4	10.9	23.4
CT24-215	38.9	39,9	27.5	23.3
CT24-216	31.6	29,5	19.2	0
CT24-217	27.5	8.81	12.9	0
CT24-218	41.9	24.4	23.3	3.6
CT24-219	41.9	26,9	24.7	0
CT24-220	27.9	17.2	12.9	0
CT24-216*	9.3	9,3	0	1
CT24-234	49.5	48.4	41	41
CT24-214*	18.7	3.3	5,5	1.1

(*) indicate animals that were recaptured and resampled. ID: identification.
